# C-752-06. Propensity-Matched Cohort Comparison: Decellularized Fish Xenograft Shows Comparable Outcomes to Allograft in Burn Site Preparation

**DOI:** 10.1093/jbcr/irag033.019

**Published:** 2026-04-06

**Authors:** Piper Schneider, Keith R Sweitzer, Julia Chrisbacher, Derek Bell

**Affiliations:** University of Rochester Medical Center, Rochester, NY; University of Rochester Medical Center, Rochester, NY; University of Rochester Medical Center, Rochester, NY; University of Rochester Medical Center, Rochester, NY

## Abstract

**Introduction:**

The use of allograft for preparation of burn sites prior to definitive grafting is a common modality in burn wound management. In recent years, the introduction of alternative dressings, such as biosynthetic and xenograft materials, has expanded the available options for optimizing wound preparation. This study compares clinical outcomes and costs between allograft and decellularized fish integument xenograft in the treatment of burn injuries.

**Methods:**

A retrospective review was conducted of patients treated with decellularized fish xenograft (n = 42) or allograft (n = 46) from July 2022–2025. Allograft patients were propensity-matched to create comparable cohorts. Demographics and outcomes including age, BMI, wound size, cost, time to autografting, regrafting, debridements, and revisions, were analyzed using unpaired t-tests and Mann–Whitney U testing. Cost analysis used unit prices of $27.00/cm2 for decellularized fish xenograft and $2.20/cm2 for allograft.

**Results:**

Patients receiving decellularized fish xenograft had a mean age of 57.5 ± 19.6 years with BMI 28.6 ± 9.7. The matched allograft group averaged 46.8 ± 24.7 years with BMI 28.1 ± 6.4. The groups did not differ in wound size (293.7 ± 479.3 cm2 vs 173.8 ± 220.1 cm^2^; t(86) = -1.5, *p*=.13), however median cost was significantly higher for xenograft ($2673.00) compared to allograft ($312.40; U = 125, *p*<.0001). There were no statistically significant differences between the decellularized fish xenograft and allograft groups for time to definitive autografting (20.8 ± 12.5 days vs. 20.3 ± 18.5 days; t(86) = 0.13, *p*=.90) (Fig. 1a), times regrafted (0.07 ± 0.3 vs. 0.04 ± 0.2; t(86) = 0.56, *p*=.58) (Fig. 1b), number of debridements (0.1 ± 0.4 vs. 0.04 ± 0.2; t(86) = 0.82, *p*=.41) (Fig. 1c), or revision procedures (0 vs. 0.02 ± 0.2; t(86) = -0.95, *p*=.34) (Fig. 1d).

**Conclusions:**

Decellularized fish xenograft was associated with no differences in time to autografting, regrafting, debridements, or revision procedures compared to allografting, but significantly increased cost.

**Applicability of Research to Practice:**

These findings highlight that although decellularized fish xenograft is clinically an equally effective alternative to allografting, the significantly higher cost supports allograft as a more practical choice in the preparation of burn wound sites for definitive autografting.

**Funding for the study:**

N/A.

